# Dental Colleges

**Published:** 1861-05

**Authors:** 


DENTAL COLLEGES.—The number of students in attendance at the Pennsylvania College of Dental Surgery during the session of 1860–61 was 63, 36 of whom were graduated. The class of the Baltimore College of Dental Surgery numbered 58, and the degree of "Doctor of Dental Surgery" was conferred upon 29 candidates. Five students were also graduated at the Ohio College of Dental Surgery. We learn that a dental college is about to be established at New Orleans.

